# A novel SIGGW dual post band-pass filter for 5G millimeter-wave band applications with a transmission zero

**DOI:** 10.1038/s41598-023-47490-1

**Published:** 2023-11-25

**Authors:** Kamran Salehian, Majid Tayarani

**Affiliations:** Iran University of Science and Technology,School of Electrical Engineering, Tehran, 1684613114 Iran

**Keywords:** Electrical and electronic engineering, Materials for devices, Electronics, photonics and device physics

## Abstract

Integration of planar circuits been considered a credible technique for low-cost mass production of microwave and millimeter-wave circuits and systems. For the first time, in this research a dual-post band-pass filter is designed and simulated in a three-layer substrate integrated gap groove waveguide (SIGGW) for 5G millimeter-wave frequency band applications. The filter includes 12 posts ($6 \times 2$). Also, the structure facilitates to use resonant posts and so we can design the posts to add a transmission zero in lower rejection band. The design theory algorithm and its limitations are investigated based on the circuit model of filter. The results shows that FBW of 5% and a lower band transmission zero for the proposed 12 posts filter. Also, the results are verified by simulation using CST. According to the results, the proposed filter is a good option for Ka-band applications and can be used as the building block for suppressing the LO leakage that is commonly used for up-converting the 5G signal to Ka band.

## Introduction

**Figure 1:**
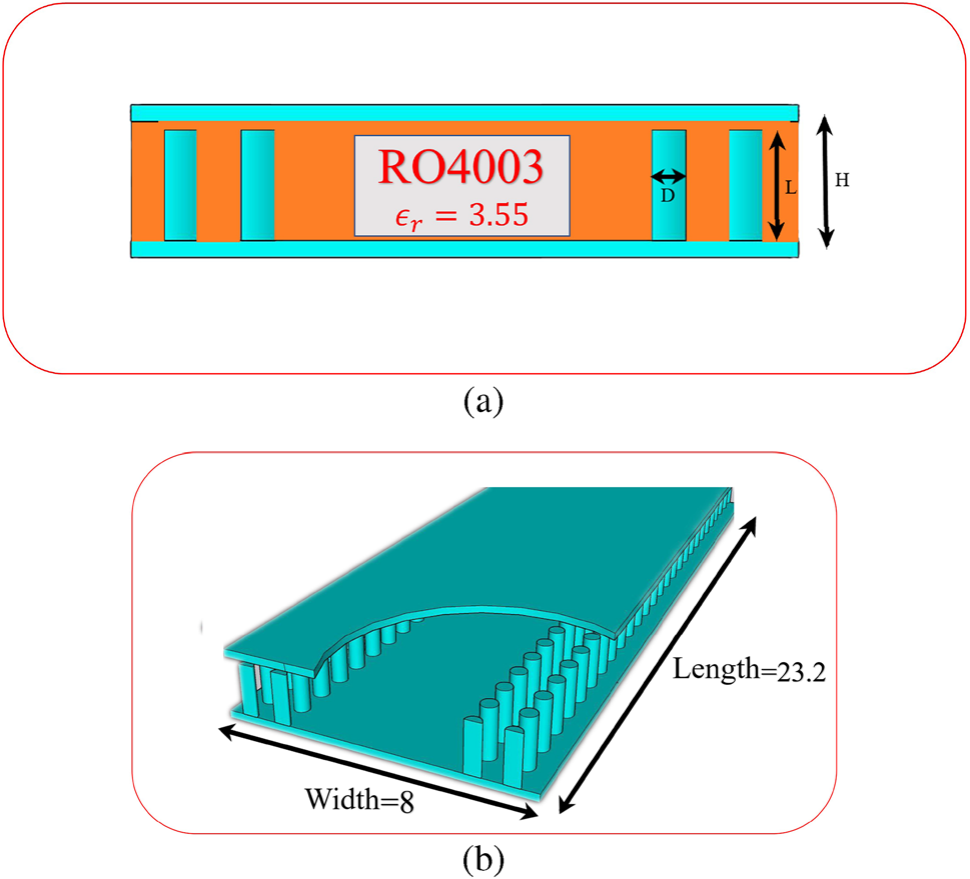
The geometry of SIGGW. (**a**) Front view of structure with RO4003 (**b**) Perspective view of SIGGW without dielectric.

The microwave bandpass filters are one of the most important building blocks of microwaves and millimeter wave communication systems. Waveguide and printed filters are known as the most common technologies, with their advantages and disadvantages well-discussed^1^. Several technologies, such as low temperature co-fired ceramic (LTCC)^2–4^ ,microstrips^5–7^, waveguides^8,9^, and micro-electromechanical systems (MEMS)^10,11^, are used to develop bandpass filters with specific characteristics such as low cost, high selectivity, low insertion loss, broad bandwidth, compact size, and power handeling capacity. Due to the trade-off between these parameters, sometimes we have to prioritize a number of characteristics that are more important for our design. Several other technologies have been introduced for the specific reason of integrating different technologies in order to use their advantages together. Substrate integrated waveguide (SIW) is one the most familiar ones, which has found a lot of applications such as low production costs, high quality factor, and ability of integration specially, specially in millimeter-waves bands^12–15^. Gap waveguides introduced in^16^ is another new technology with some advantages comparing with classic waveguides. The gap waveguide can be a low-loss building block with advantages in manufacturig waveguide components such as antennas, filters, couplers, and also integration of active components like amplifiers and microwave monolithic integrated circuits (MMIC’s) at millimeter-waves and THz bands^17,18^. These structures are based on two parallel plates, with a bed of pins structure in one of the plates (see Fig. 1). This bed of pins structure introduces a high impedance condition over this plate that avoids the electrical contact requirement between the top plate and the pins of the bottom plate. The direction of the wave propagation relative to the direction of corrugation determines whether it enables or prevents the wave propagation at the surface^19^.

Recently, manufacturing problems in mm-wave and THz designs motivated consideration of the gap waveguide technology^20–22^. Common types of gap waveguide technologies are groove gap waveguide (GGW)^23^, microstrip gap waveguide (MGW)^24^ ridge gap waveguide (RGW)^25^ and substrate integrated groove gap waveguide (SIGGW)^26,27^. SIGGW is used in this research to design a band-pass filter at Ka band with a transmission zero.

In section 2, we briefly explain the SIGGW design for Ka-Band. In Sect. 3, we analytically design the BPF using dual-post configuration. We also explain how to use the out of band resonance for the SIGGW posts to realize a transmission zero in lower rejection band. Section 4 is used for simulation of structure and verifying the results and compare with calculated response at third section. Finally, we present a design method, followed by some concluding remarks.

## Substrate integrated groove gap waveguide (SIGGW) structure

The geometry of the SIGGW is shown in Fig. 1. Unlike classic waveguides, the SIGGW doesn’t require electrical contact between the upper and lower planes. The dimensions and positions of metallic bars are designed to create a metamaterial gap at desired frequency band to prevent propagation in the x-direction and virtually act as a wall in that band. As a result, EM wave propagates through the space between top plane, metal pins, and lower plane which is filled with a dielectric. Here, we use RO4003 with $\epsilon _r$=3.55 and $tan\delta$=0.002 as dielectric. The dimensions of metal pins are designed to achieve a band gap around 28 GHz^25^. The designed parameters are depicted in Table 1.

**Table 1:** Desired parameter for design SIGGW. $T_x$ and $T_y$ are distance center to center of metal pins and D is diameter of metal pins.

Parameter	Value (mm)
Width	8
Length	20
H	1.778
L	1.542
$T_x$	1.05
$T_y$	1
G=H-L	0.236
D	0.5

**Figure 2:**
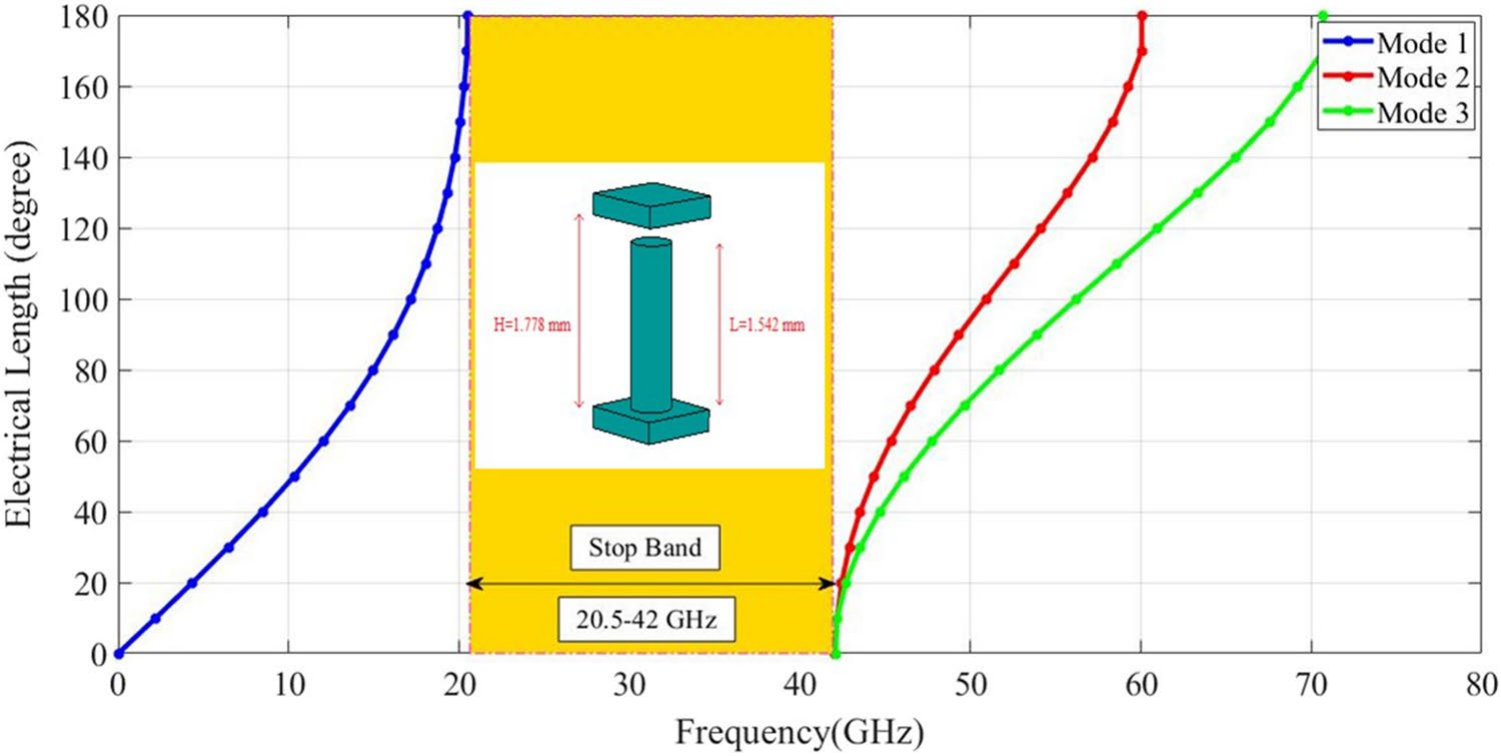
The bandgap between first and second mode of excitation.

**Figure 3:**
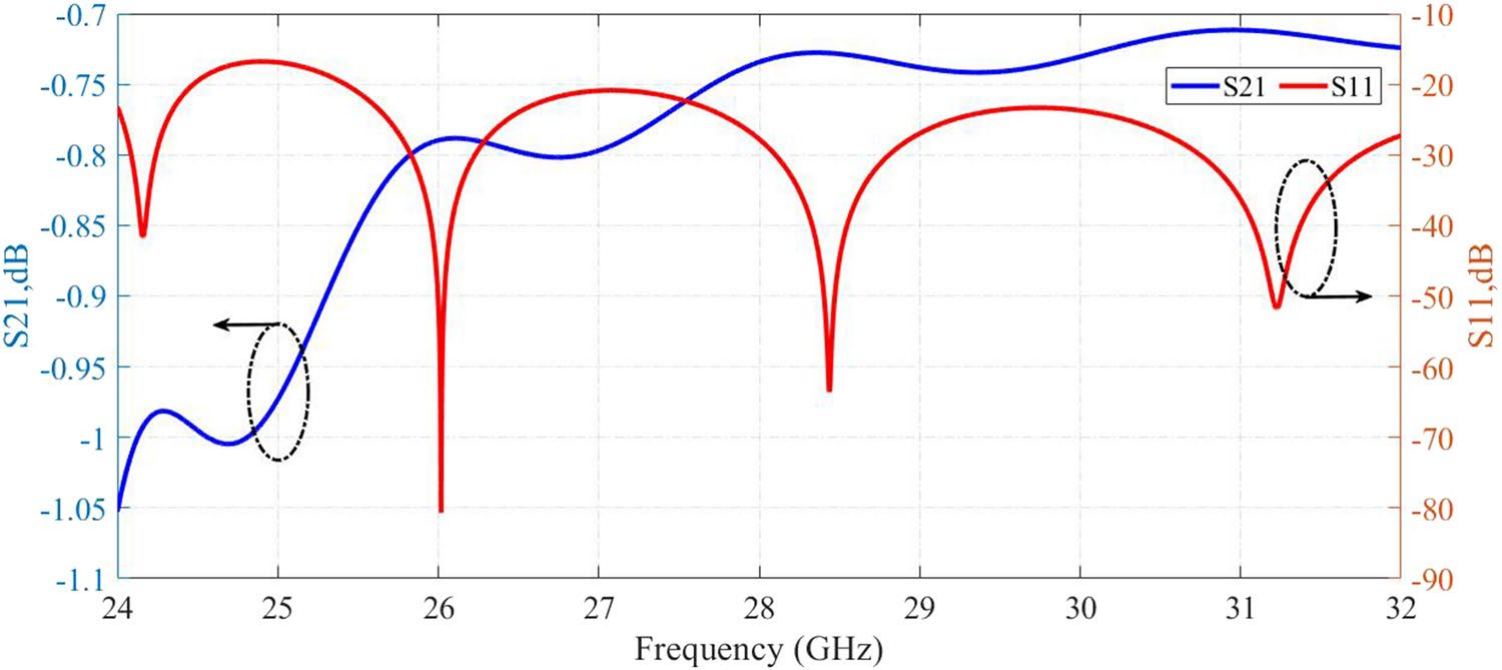
Return loss and transmission coefficient of designed SIGGW.

The simulated results for values in Table 1 are shown in Fig. 2. The dispersion curve indicates that the first bandgap is between 20.5GHz to 42GHz. In Fig. 3, the s-parameters of the designed SIGGW are displayed, revealing excellent return loss and transmission coefficients in the range of 24GHz-32GHz.

## Dual post filter design

**Figure 4:**
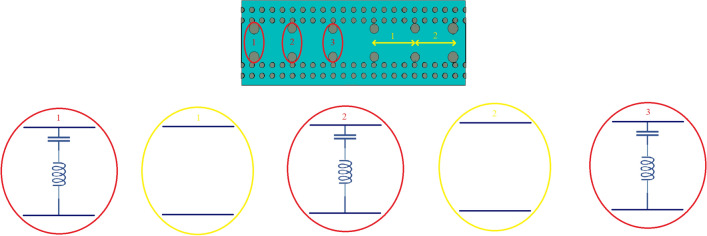
Equivalent circuit of filter.

One of candidates for band-pass waveguide filter is inductive dual-post structure^28^. In section 3, we designed the SIGGW, and in this section, we use those results to design our filter. The filter is designed at center frequency of 28GHz and fractional bandwidth of about 5 We use posts at distance about $\frac{\lambda _g}{2}$ from each other, where $\lambda _g$ is guided wavelength at center frequency, taking into consideration the $\epsilon _r$ of structure. For designing dual-post filters in classic rectangular waveguide, we use posts connecting bottom to top of the waveguide, but in this case the posts are shorter than the height of SIGGW. The post lengths, denoted as L from the bottom, as shown in Fig. 1. Since the L is shorter than waveguide height, there will be a gap between the top of the posts and the upper plate. A capacitance is formed between the posts and upper conductor. This can be so useful to generate a transmission zero in rejection band of filter. The posts are inductive and having a capacitance in series cause a resonance frequency which short circuit the SIGGW and current is maximum in this case. Nevertheless, we need inductances for our filter topology and fortunately, as the frequency increases, the behavior of series inductance and capacitance becomes more inductive, so in this case we expect that if the frequency increases this design behaves perfectly as an inductive post. This means we have a resonant frequency at lower rejection band that could be useful if we can control it at proper frequency. So here, we introduce an algorithm to control this transmission zero. The equivalent circuit of waveguide filter is shown in Fig. 4.

The ABCD matrix and input impedance of transmission line is given by Eqs. (1) and (2) respectively.

1 $$\begin{aligned}{}[ABCD]= & {} \begin{bmatrix} cos\beta l &{} jZ_0 sin\beta l \\ jY_0 sin\beta l &{} cos\beta l \\ \end{bmatrix} \end{aligned}$$

2 $$\begin{aligned} Z_{in}= & {} Z_0\frac{Z_l+jZ_0 tan\beta l}{Z_0+jZ_l tan\beta l} \end{aligned}$$

**Figure 5:**
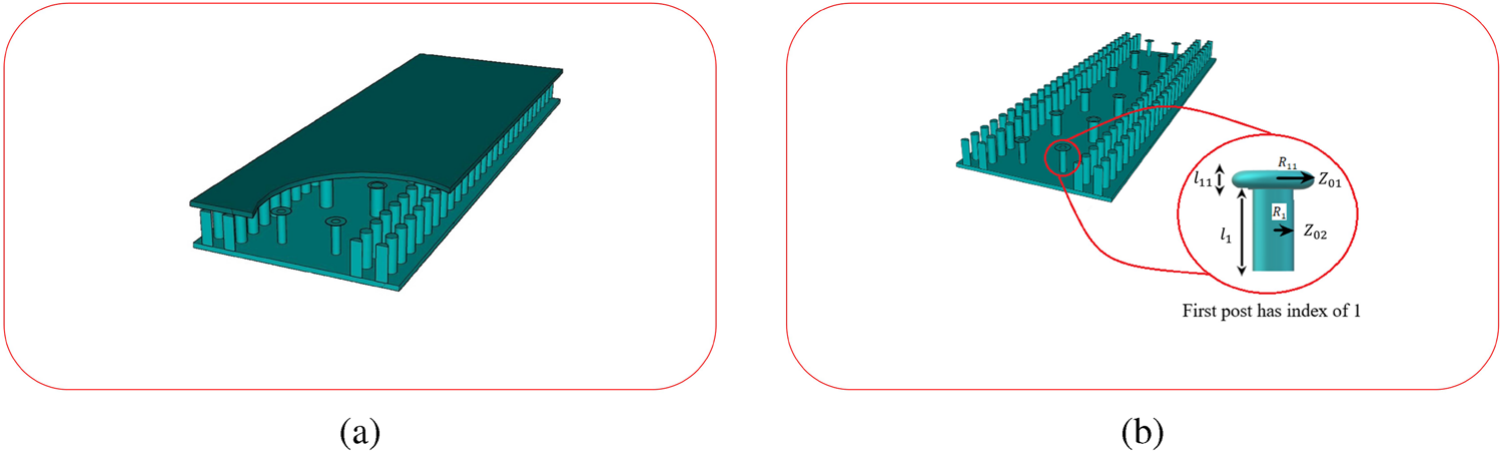
Final structure of filter. (**a**) Perspective view of SIGGW with cuting plane (**b**) Enlarged view of posts.

If $Z_l\rightarrow \infty$ the Eq. (2) becomes $Z_{in}=-jZ_{01} cot\beta l_{11}$. So, the input impedance of structure is Eq. (3) (see Fig. 5).

3 $$\begin{aligned} Z_{in}=Z_{02}\frac{-jZ_{01} cot\beta l_{11}+jZ_{02} tan\beta l_1}{Z_{02}+j(-jZ_{01} cot\beta l_1)tan\beta l_1}=jZ_{02}\frac{x tan\beta l_1-cot\beta l_{11}}{x+cot\beta l_{11} tan\beta l_1} \end{aligned}$$

where, $$x=\frac{Z_{02}}{Z_{01}}$$ so: $$Y_{in}=jY_{02}\frac{x+cot\beta l_{11} tan\beta l_1}{x tan\beta l_1-cot\beta l_{11}}$$

We mentioned that distance between posts is about $\frac{\lambda _g}{2}$ so the ABCD matrix of total structure is Eq. (4).

4 $$\begin{aligned} {[}T] = \begin{bmatrix} cos\beta l &{} jZ_0 sin\beta l \\ jY_0 sin\beta l &{} cos\beta l \\ \end{bmatrix} \times \begin{bmatrix} 1 &{} 0 \\ jY_{02}\frac{x+cot\beta l_{11} tan\beta l_1}{x tan\beta l_1-cot\beta l_{11}} &{} 1 \\ \end{bmatrix} \times \begin{bmatrix} cos\beta l &{} jZ_0 sin\beta l \\ jY_0 sin\beta l &{} cos\beta l \\ \end{bmatrix} \end{aligned}$$

where $Z_0$ and $\beta l$, is characteristic impedance and electrical length of resonators respectively. If $l_{11}<\lambda /4$ from taylor series we have $cot \beta l_{11}\approx 1$ due to Fig. 5 it is a reasonable assumption. As a result, with $\beta l=\pi$:

$$\begin{aligned} {[}T] = \begin{bmatrix} -1 &{} 0 \\ 0 &{} -1 \\ \end{bmatrix} \times \begin{bmatrix} 1 &{} 0 \\ jY_{02}\frac{x+\frac{tan \beta l_1}{\beta l_{11}}}{x tan\beta l_1-\frac{1}{\beta l_{1_1}}} &{} 1 \\ \end{bmatrix} \times \begin{bmatrix} -1 &{} 0\\ 0 &{} -1\\ \end{bmatrix} = \begin{bmatrix} 1 &{} 0 \\ jY_{02}\frac{x\beta l_{11}+tan \beta l_1}{x\beta l_{11} tan \beta l_{11}} &{} 1 \\ \end{bmatrix} \end{aligned}$$

If we convert *ABCD* matrix to S-matrix due to^29^:

$$\begin{aligned}{} & {} S_{11}=\frac{A+\frac{B}{Z_0}-C Z_0-D}{A+\frac{B}{Z_0}+C Z_0+D}\qquad \qquad S_{12}=\frac{2}{A+\frac{B}{Z_0}+C Z_0+D}\\{} & {} S_{11}=S_{22}=\frac{(x \beta l_{11} tan \beta l_1 -1)}{2(x \beta l_{11} tan \beta l_1 - 1)+j(x \beta l_{11} + tan \beta l_1)} \end{aligned}$$

At resonance frequency we choose $|S_{11}|=0$ and $|S_{21}|=1$.

5a $$\begin{aligned} x \beta l_{11} + tan\beta l_1&=0 \end{aligned}$$

5b $$\begin{aligned} \frac{(x \beta l_{11} tan \beta l_1 -1)}{\sqrt{{4(x \beta l_{11} tan \beta l_1 - 1)^2+j(x \beta l_{11} + tan \beta l_1)^2}}}&=1 \end{aligned}$$

In this case we have Quasi-TEM mode so we compute inductance and capacitance of posts. It is assumed that the inductance of $l_1$ and capacitance of $l_{11}$ are dominant. First, we compute inductance, form Ampere law we have:

$$\begin{aligned} \oint _{C} H.\,dl= & {} I {\rightarrow } H=\frac{I}{2 \pi r} \\ \frac{\mu }{2} \iiint \ |H^2|\ dv= & {} \frac{\mu }{2} \iiint \ \frac{I^2}{4\pi ^2 r^2}\,rdrd\phi dz = \frac{\mu I^2}{4 \pi } \int \limits _{R_1}^{R_{11}} \frac{1}{r}. \, dr \int \limits _{0}^{l_{1}} \, dz = \frac{\mu I^2}{4\pi } l_1 \ln {\frac{R_{11}}{R_1}} \end{aligned}$$

On the other hand, we know the energy of inductance is W=$\frac{1}{2}LI^2$ so: $L=\frac{\mu }{2 \pi }l_1 \ln {\frac{R_{11}}{R_1}}$

Second, we compute capacitance. $C=\epsilon _r \frac{A}{G}$ $\rightarrow$ $C=\frac{3.55}{36 \pi \times 10^9}\frac{\pi R_{11}^2}{G}$

So, the resonance frequency due to the post is:

6 $$\begin{aligned} \omega _c=\frac{1}{\sqrt{LC}}=\frac{1}{\sqrt{{\frac{\mu }{2 \pi } l_1 \ln {\frac{R_{11}}{R_1}}}\times \frac{3.55}{36\pi \times 10^9}\frac{\pi R_{11}^2}{G}}} \end{aligned}$$

Electric field and magnetic field are minimum and maximum respectively on posts, so assuming that the waveguide is strip line the radius of $R_1$ and $R_2$ related to $Z_{01}$ and $Z_{02}$. The impedance $Z_0$ of a transmission line consisting of a circular signal conductor having a diameter *D* and centered between parallel ground planes separated by distance *T* is given by Eq. (7)^30^.

7 $$\begin{aligned} Z_0=\frac{\eta _0}{2\pi \sqrt{\epsilon _r}} \ln {\frac{4T}{\pi D}} \end{aligned}$$

Algorithm of design:

1. Choose the frequency that want to have transmission zero out of band and compute $\omega _c$
2. From Eq. (7) compute $R_{11}$ and $R_1$ according to $Z_{01}$ and $Z_{02}$.
3. Now we have 3 equations and 4 unknowns. (3 equations are Eq. 5a,  b and Eqs. (6) and (4) unknowns are $Z_{01}$, $Z_{02}$, $l_{11}$, $l_1$)
4. By assuming that one parameter is constant, compute other parameters. (For example, initially set $l_1$ around $\frac{\lambda _g}{4}$ and compute $Z_{01}$, $Z_{02}$, $l_{11}$).

**Figure 6:**
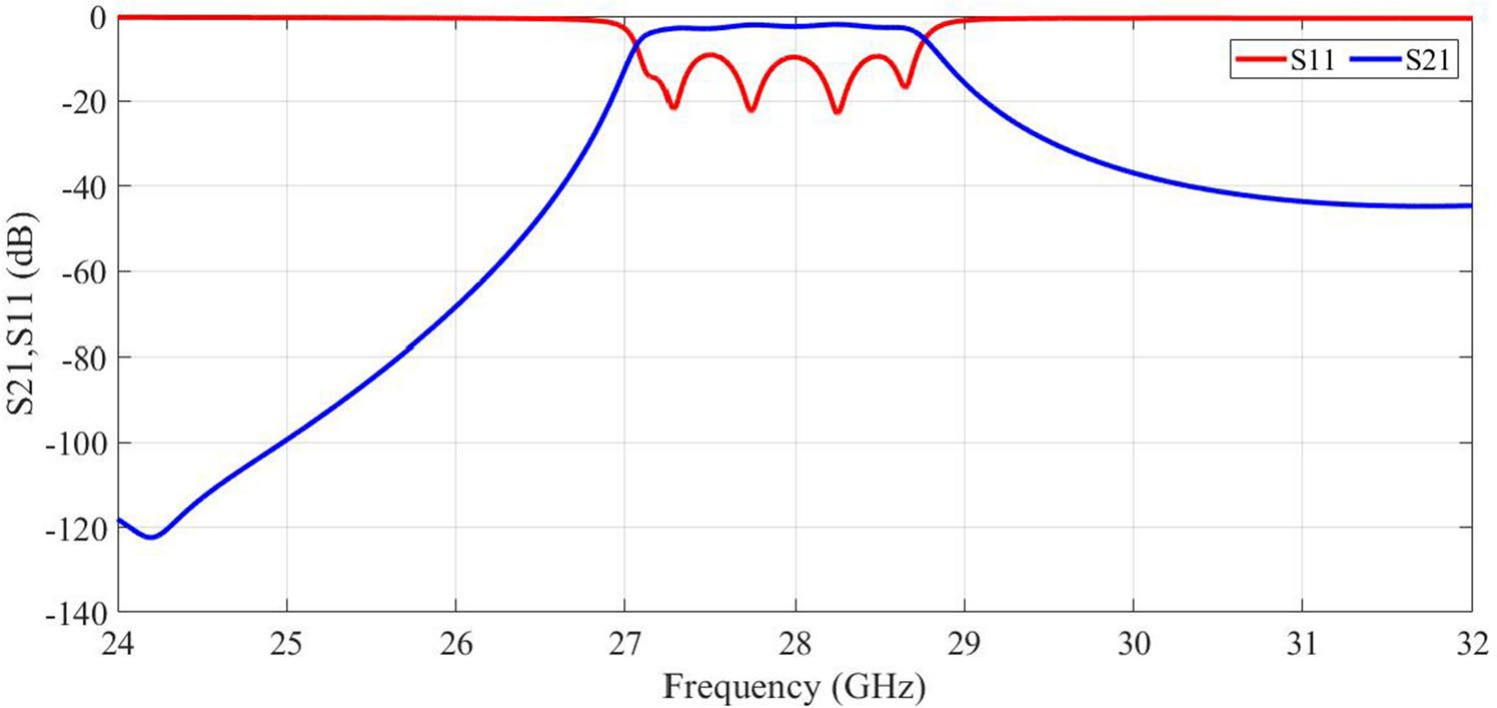
Final result of filter.

The final design parameters are depicted in Table 2 We must mention that there are limitations to this structure. At lower frequencies, designing and building this structure will become difficult due to the increase in substrate thickness. Similarly, at higher frequencies, it will also be challenging to design and build this structure because of the shrinking dimensions of the filter.The final structure and results are shown in Figs. 5 and 6 respectively.

**Table 2:** Design parameters for posts. Index 1 and 11 is for the first post, index 2,22 and 3,33 is for second and third posts respectively.

Parameter	Value (mm)
$l_1$	1.524
$l_{11}$	0.018
$l_2$	1.524
$l_{22}$	0.018
$l_3$	1.524
$l_{33}$	0.018
$R_{11}$	0.54
$R_{22}=R_{33}$	0.47
$R_1$	0.2
$R_2=R_3$	0.3

## Simulation of SIGGW filter

**Figure 7:**
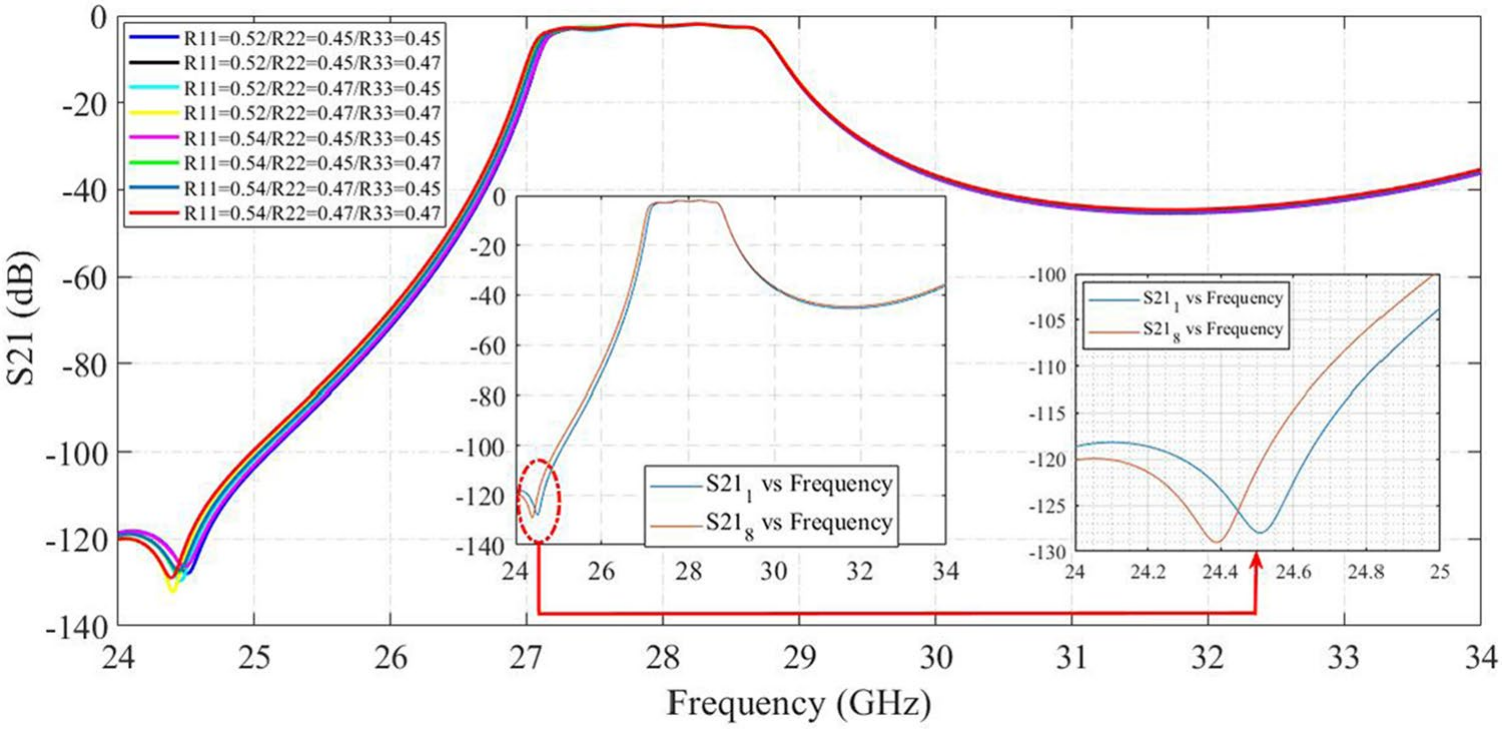
Effect of changing the area of capacitors in the location of transmission zero.

For verifying our results, we use sweep parameters in CST. If we reduce the radius of capacitors from Eq. (6), we see that resonance frequency is increased. It can be seen from Fig. 7 that if radius of each capacitor is decreased about 0.02 mm the resonance frequency increased about 100MHz.

**Figure 8:**
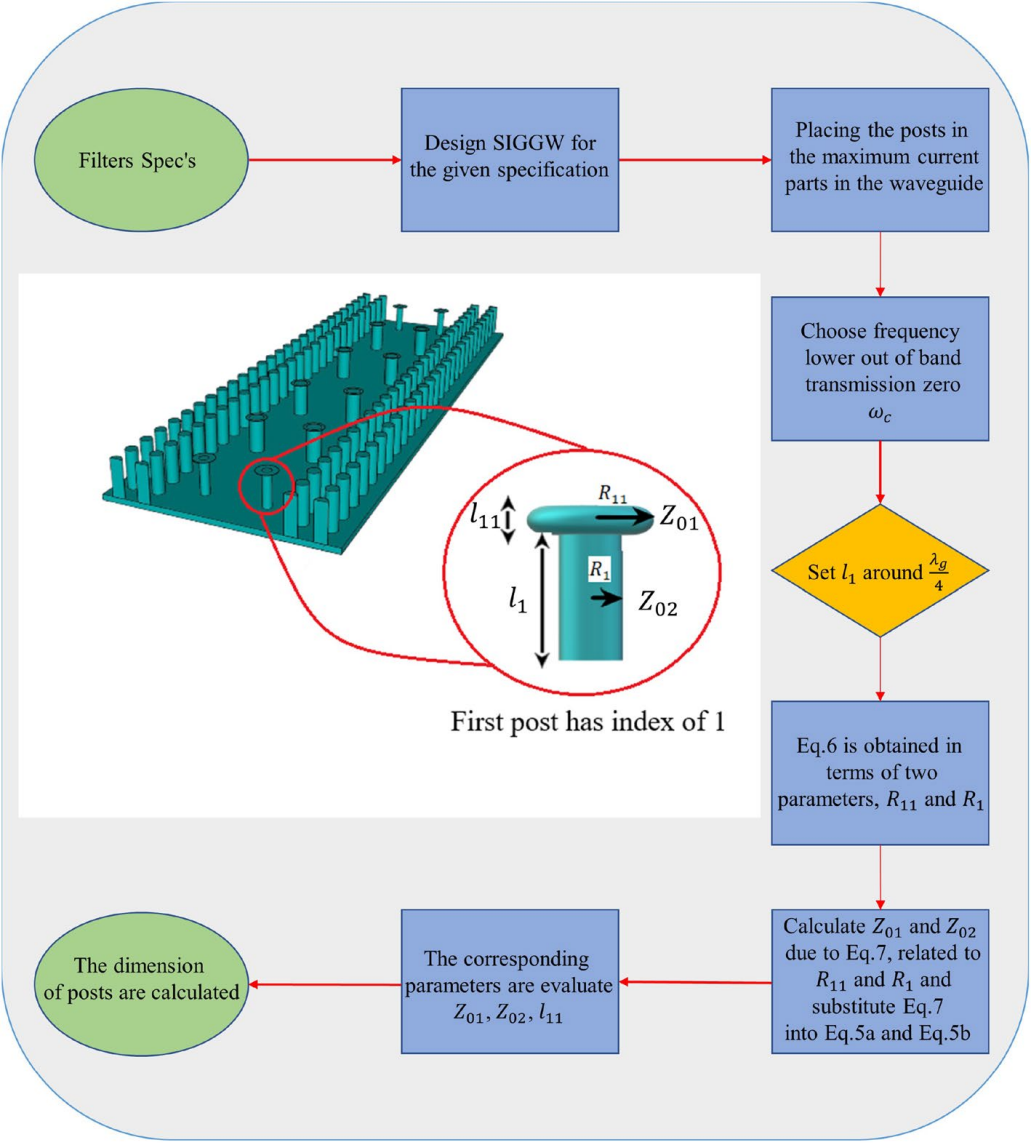
Flow chart of the main steps in the SIGGW filter design with finite transmission zero in lower band.

Figure 8 illustrates the primary objective of this paper, which is to present the design of the SIGGW filter in the Ka band with finite transmission zero in the lower band. This analysis method can also be employed for designing other resonant filters that incorporate finite transmission zero.

## Discussion

In this research, a dual-post band-pass filter in the 5G Ka-band has been designed and simulated using a three-layer substrate integrated gap groove waveguide (SIGGW). After interpreting the dual-post filters, we proceeded with the SIGGW filter design. The most crucial advantage of gap waveguide structures compared to conventional rectangular waveguides is that the posts are not connected to the top wall of the waveguide. In SIGGW, the multilayer PCB etching and plating technology forces us to provide some pads on both sides of the via holes. These pads play the role of capacitor plates when we realize the posts as blind vias. We have shown that this can be efficiently controlled and facilitates the utilization of the resonant frequency of the filter posts to synthesize a transmission zero at a lower reject band. The realized transmission zero improves the lower side rejection slope and can be used for suppressing the LO leakage, which is commonly employed for up-converting the 5G signal to the Ka-band. We have proposed an algorithm to control this transmission zero, verified using CST simulating software. This algorithm can create transmission zero in other gap waveguide resonant filters and obtain a suitable initial solution. The results agree with the introduced simple circuit model for the resonant frequency. Finally, Table 3 compares the BPFs that were recently reported and fabricated using different technologies (the mass of the construction was not included due to a lack of available data).

**Table 3:** Performance comparisons between proposed filter in this paper with other filters in different technologies.

References	Technology	Order	Center frequency (GHz)	FBW ($\%$)	I.L (dB)	Size ($\lambda _0 \times \lambda _0 \times \lambda _0$)	Material	$\epsilon _r$
[^31^]	RGW	3	35	1	1	2.08$\times$1.88$\times$1.48	Metal	–
[^32^]	RGW	2	15	4.5	1	3.23$\times$3.23$\times$0.35	Metal	–
[^33^]	GGW	4	35.65	1.4	0.5	2.49$\times$2.49$\times$0.36	Metal	–
[^34^]	GGW	5	14	1	1.22	4.72$\times$1.11$\times$0.34	Metal	–
[^35^]	GGW	4	12.1	2.4	0.3	2.23$\times$1.65$\times$0.4	Metal	–
[^36^]	GGW	6	11.9	3.88	0.5	2.64$\times$2.31$\times$0.38	Metal	–
[^37^]	SIW	2	13.9	3.5	1.8	1.1$\times$0.46$\times$0.03	Dielectric	3.5
[^28^]	SIW	5	8.2	6	2.5	2.23$\times$0.4$\times$0.03	Dielectric	2.6
[^27^]	SW-SIGGW	2	12	3.2	1.3	0.52$\times$0.29$\times$0.07	Dielectric	2.2
**This Work**	**SIGGW**	**5**	**28**	**5**	**1**.**8**	**2**.**16**$\times$**0**.**74**$\times$**0**.**16**	**Dielectric**	**3**.**55**

## Data Availability

The data that backs up the conclusions of this research can be obtained from M.T. and K.S., but there are limitations on accessing this data. The data used in this study were obtained under a license and are not accessible to the public. However, the authors can provide the data upon a reasonable request with permission from M.T.

## References

[1] Cameron, R.J., Kudsia, C.M. & Mansour, R.R. Microwave filters for communication systems: fundamentals, design, and applications (John Wiley & Sons, 2018).

[2] Zhang XY (2014). Compact LTCC bandpass filter with wide stopband using discriminating coupling. IEEE Trans. Componen. Packag. Manuf. Technol..

[3] Xu J-X, Zhang XY, Zhao X-L, Xue Q (2016). Synthesis and implementation of LTCC bandpass filter with harmonic suppression. IEEE Trans. Compon. Packag. Manuf. Technol..

[4] Zheng Y, Sheng W (2017). Compact lumped-element LTCC bandpass filter for low-loss vhf-band applications. IEEE Microw. Wirel. Compon. Lett..

[5] Fallahzadeh S, Tayarani M (2010). A new microstrip UWB bandpass filter using defected microstrip structures. J. Electromagn. Waves Appl..

[6] Zhang W, Gu J, Xu G, Luo L, Li X (2020). Copper/benzocyclotene thin film technique based microstrip bandpass filter featured by thick dielectric layer for low insertion loss. Microw. Opt. Technol. Lett..

[7] Aristarkhov, G., Kirillov, I., Arinin, O., Markovskiy, A. & Doronina, A. Multi-band bandpass microstrip filters based on two codirectional hairpin resonators. In 2023 Systems of Signals Generating and Processing in the Field of on Board Communications 1–5 (2023).

[8] Fallahzadeh S, Bahrami H, Tayarani M (2009). A novel dual-band bandstop waveguide filter using split ring resonators. Progress. Electromagn. Res. Lett..

[9] Melgarejo JC (2023). A new family of reconfigurable waveguide filters and diplexers for high-power applications. IEEE Access.

[10] Shim Y, Wu Z, Rais-Zadeh M (2012). A high-performance continuously tunable mems bandpass filter at 1 GHZ. IEEE Trans. Microw. Theory Tech..

[11] Dey S, Koul SK (2020). Reliable, compact, and tunable mems bandpass filter using arrays of series and shunt bridges for 28-GHZ 5g applications. IEEE Trans. Microw. Theory Tech..

[12] Zhang Q-L, Yin W-Y, He S, Wu L-S (2010). Compact substrate integrated waveguide (SIW) bandpass filter with complementary split-ring resonators (CSRRS). IEEE Microw. Wirel. Compon. Lett..

[13] Mahan A, Pourjafari SM, Tayarani M, Mahani MS (2019). A novel compact dual-mode SIW filter with wide rejection band and selective response. Microw. Opt. Technol. Lett..

[14] Röhrl, F. X. *et al.* Cost-effective SIW band-pass filters for millimeter wave applications a method to combine low tolerances and low prices on standard PCB substrates. In 2017 47th European Microwave Conference (EuMC) 416–419 (2017).

[15] Guo Y-J, Xu K-D, Deng X, Cheng X, Chen Q (2020). Millimeter-wave on-chip bandpass filter based on spoof surface plasmon polaritons. IEEE Electron. Device Lett..

[16] Kildal P-S, Alfonso E, Valero-Nogueira A, Rajo-Iglesias E (2008). Local metamaterial-based waveguides in gaps between parallel metal plates. IEEE Antennas Wirel. Propag. Lett..

[17] Alfonso, E., Zaman, A.U., Pucci, E. & Kildal, P. S. Gap waveguide components for millimetre-wave systems: Couplers, filters, antennas, mmic packaging. In *2012 International Symposium on Antennas and Propagation (ISAP)* 243–246 (2012).

[18] Rajo-Iglesias E, Ferrando-Rocher M, Zaman AU (2018). Gap waveguide technology for millimeter-wave antenna systems. IEEE Commun. Mag..

[19] Kildal P-S, Zaman AU, Rajo-Iglesias E, Alfonso E, Valero-Nogueira A (2011). Design and experimental verification of ridge gap waveguide in bed of nails for parallel-plate mode suppression. IET Microw. Antennas Propag..

[20] Ali MMM, Shams SI, Elsaadany M, Gagnon G, Wu K (2022). Graphene-based terahertz reconfigurable printed ridge gap waveguide structure. Sci. Rep..

[21] Farjana, S. *et al.* Multilayer dry film photoresist fabrication of a robust> 100 GHZ gap waveguide slot array antenna. *IEEE Access* (2023).

[22] Hongjian W, Min Y, Minzheng M (2023). Terahertz groove gap waveguide leaky wave antenna. Microw. Opt. Technol. Lett..

[23] Rajo-Iglesias, E. & Kildal, P. S. Groove gap waveguide: A rectangular waveguide between contactless metal plates enabled by parallel-plate cut-off. In *Proceedings of the Fourth European Conference on Antennas and Propagation*, 1–4 (2010).

[24] Brazalez AA, Rajo-Iglesias E, Vazquez-Roy JL, Vosoogh A, Kildal P-S (2015). Design and validation of microstrip gap waveguides and their transitions to rectangular waveguide, for millimeter-wave applications. IEEE Trans. Microw. Theory Tech..

[25] Alfonso, E. *et al.* Design of microwave circuits in ridge-gap waveguide technology. In *2010 IEEE MTT-S International Microwave Symposium*, 1544–1547 (2010).

[26] Deng J-Y (2020). Slow-wave substrate integrated groove gap waveguide. IEEE Microw. Wirel. Compon. Lett..

[27] Deng J-Y (2021). Ultracompact bandpass filter based on slow wave substrate integrated groove gap waveguide. IEEE Trans. Microw. Theory Tech..

[28] Adabi A, Tayarani M (2008). Substrate integration of dual inductive post waveguide filter. Progress Electromagn. Res. B.

[29] Pozar, D. M. *Microwave engineering* (John wiley & sons, 2011).

[30] Nguyen, C. *Analysis methods for RF, microwave, and millimeter-wave planar transmission line structures* (John Wiley & Sons, 2003).

[31] Ahmadi B, Banai A (2015). Direct coupled resonator filters realized by gap waveguide technology. IEEE Trans. Microw. Theory Tech..

[32] Chhasatia, N. J., Chaudhari, J. P. & Patel, A. V. Ridge gap waveguide based band pass filter for ku-band application. In *IOP Conference Series: Materials Science and Engineering*, vol. 1206, 012011 (2021).

[33] Al-Juboori B (2018). Lightweight and low-loss 3-d printed millimeter-wave bandpass filter based on gap-waveguide. IEEE Access.

[34] Zaman AU, Kildal P-S, Kishk AA (2012). Narrow-band microwave filter using high-q groove gap waveguide resonators with manufacturing flexibility and no sidewalls. IEEE Trans. Compon. Pack. Manuf. Technol..

[35] Máximo-Gutiérrez C, Hinojosa J, Alvarez-Melcon A (2023). Design of evanescent mode band-pass filters based on groove gap waveguide technology. AEU-Int. J. Electron. Commun..

[36] Liu Z, Deng J-Y, Sun D (2019). Slow-wave groove gap waveguide bandpass filter. IEEE Access.

[37] Annadurai BP, Hyder Ali UH (2020). A compact SIW bandpass filter using DMS-DGS structures for ku-band applications. Indian Acad. Sci..

